# The impact of coronavirus lockdown on oral healthcare and its associated issues of pre-schoolers in China: an online cross-sectional survey

**DOI:** 10.1186/s12903-021-01410-9

**Published:** 2021-02-06

**Authors:** Chang Liu, Shuang Zhang, Chenzheng Zhang, Baojun Tai, Han Jiang, Minquan Du

**Affiliations:** The State Key Laboratory Breeding Base of Basic Science of Stomatology (Hubei-Most) and Key Laboratory for Oral Biomedical Engineering of Ministry of Education, School and Hospital of Stomatology, Wuhan University, 237 Luoyu Road, Wuhan, 430079 China

**Keywords:** Coronavirus, Lockdown, Preschool children, Oral health

## Abstract

**Background:**

The sudden outbreak of coronavirus disease (COVID-19) epidemic influenced people’s daily life. During lockdown of Wuhan city, the oral health and its associated issues of preschool children were investigated and guidance for dental clinics when the epidemic were controlled in the future were also provided.

**Methods:**

A national online survey was conducted among preschool children and completed by their caregivers. The questionnaire related to children’s oral health status and care behaviour, caregivers' attitudes. The information was statistically analyzed between Wuhan residents and others residents.

**Results:**

4495 valid questionnaires were collected. In oral health status, during Wuhan lockdown, 60.8%, 35.5% and 18.3% children had self-reported dental caries, toothache and halitosis respectively. In oral health attitudes, respondents who would increase attention to oral health was more than that would decrease. In oral hygiene behaviour, compared to non-Wuhan children, the children in Wuhan became more active in brushing their teeth. In utilization of dental services in the future, less Wuhan residents would choose to have dental visit directly, 28.5% Wuhan residents and 34.7% non-Wuhan residents agreed all of procedures could be done if proper protected.

**Conclusions:**

Oral health status and associated issues of preschool children in Wuhan were significantly different from that of others during lockdown of Wuhan city and in the future. Effective measures should be taken as early as possible to protect children's oral health.

## Background

In late December 2019, the coronavirus disease (COVID-19) crisis emerged in Wuhan, China. To quickly control the spread of the epidemic, the Chinese government adopted a series of decisive measures. Wuhan, situated in the middle of China with 10 million people, was the first city in China to be quarantined on 23 January [1].

This sudden public incident not only influenced daily life but also hit all areas of health care, especially in dentistry [2]. Due to the dental aerosols generated during treatment and the close distance between dentists and patients, the dental clinic could be an environment with a risk of spreading the virus. Thus, most dental clinics suspended routine oral clinics in China, which may inevitably influence people's oral health care [3].

In China, the oral health status of preschool children is unpleasant and even worrying [4, 5]. Early childhood caries (ECC) is an international public health problem [6, 7], and accounts for the highest incidence of chronic diseases among children. It may be influenced by the unavailability of dental services during the COVID-19 epidemic [8].

Currently, with the efforts of the medical staff in the whole country and the support of all residents in Wuhan, the epidemic situation is basically under good control, and people have gradually resumed their roles in the workplace and at school. Hospitals have also provided comprehensive medical services progressively [9].

Under such circumstances, it is imperative to understand children’s oral health status and behaviour as well as caregivers’ attitude during lockdown and in the future. However, limited data have been reported recently. Therefore, in this study, we investigated the impacts of the COVID-19 lockdown on the oral health and its associated issues of preschool children in Wuhan and other areas in China and provided guidance for oral healthcare in the future.

## Methods

### Ethical aspects and participants

This study was approved by the Ethic Committee of the School and Hospital of Stomatology, Wuhan University (Approval no. HGGC-035). Caregivers with preschool children aged 3–6 years living in China during Wuhan lockdown participated in the survey. The questionnaire is completed on a voluntary basis. Completing and submitting this questionnaire would be regarded as consent to participate. In this study, random sampling was used. Accordingly, the sample size was calculated by the Cochrane formula as following. However, for more accuracy in citing the research results, the sample was increased to over 4000.

$$\begin{aligned}& {\text{Sample}}\,{\text{size}} = \left[ {{\text{X}}^{2} {\text{NP}}\left( {1 - {\text{P}}} \right)} \right]/\left[ {{\text{d}}^{2} \left( {{\text{N}} - 1} \right) + {\text{X}}^{2} {\text{P}}\left( {1 - {\text{P}}} \right)} \right] \\ & = [1.96^{2} \times 60000000 \times 0.5(1 - 0.5)\left] / \right[0.05^{2} \times (60000000 - 1) \\ & \quad + \,1.96^{2} \times 0.5(1 - 0.5)] \approx 384 \\ \end{aligned}$$

N is the population size of 3–6 years old children in China.

### Questionnaire

The questionnaire was designed by experts in preventive dentistry through discussion (Additional files 1 and 2). This cross-sectional survey was carried out from 1 to 7 May 2020, which was the week that marked 100 days after the beginning of Wuhan lockdown. Because a community-based national sampling survey was still not feasible to conduct in the present, the information was collected online. An electronic questionnaire was designed by using Questionnaire Star (http://www.wjx.cn) [10]. We conducted sampling using the snowball strategy [11]. Through the authors’ networks with people in China, a recruitment link or quick response (QR) code was distributed to groups in their WeChat accounts. Then the individuals in this group distributed to other people who can participate in the survey. This process continued until sample size met the requirements. No monetary rewards were given for the completion of the questionnaire.

The questionnaire consisted of two parts: demographics and variables. Demographics included gender (male and female), age (3, 4, 5, 6 years old), and place of residence (Wuhan, other cities of China) during Wuhan lockdown. Variables were developed according to the influences of COVID-19 on the oral health and its associated issues of preschool children. These variables addressed oral health status, attitudes and behaviour (oral hygiene and utilization of dental services behaviour).

To understand pre-schoolers’ oral health status, we listed options including dental caries, toothache, halitosis, gingival bleeding, gingival swelling, tooth trauma, fillings removal and others for them to multiply choose. It was a self-assessed question. Caregiver assessed the children whether they had these oral diseases.

Attitudes towards the oral healthcare of caregivers were also measured by questions, which were involving oral health attention and preventive measure during Wuhan lockdown and in the future when the epidemic was controlled.

In the aspect of oral health-care behaviour, there were sugar consumption behaviour, oral hygiene behaviour and utilization of dental services behaviour. For sugar consumption, three typical categories of sweet things were questioned, including sweet foods, sweet drinks, and sweetened milk/yogurt/tea/coffee. The frequencies were little or never/One to three times a month/once a week/two to six times a week/once a day/more than once a day. For oral hygiene behaviour, the questions contained: (1) brushing frequency, (2) caregiver helping brush, (3) toothpaste utilization. For utilization of dental services behaviour, the solutions of oral problems during Wuhan lockdown and in the future were compared, moreover, the worries about treatment were analyzed.

To ensure the quality of the questionnaire, a pilot survey was conducted. After the questionnaire was modified timely, the network survey was carried out on a voluntary basis. All questions were required to be completed as to ensure the response rate.

### Statistical analysis

After the national cross-sectional survey, the data were exported, logically checked and statistically analysed by SPSS 19.0 (SPSS Inc., Chicago, IL, USA) software. The results were described by the composition ratio. The differences between variables and places of residence places were compared by the chi-square test. The inspection level was set at α = 0.05.

## Results

### Demographic characteristics of preschool children

By 7 May 2020, a total of 4495 valid nationwide questionnaires from nationwide had been received, reflecting a recovery rate of 99.7%. In the survey, 2392 (53.2%) and 2103 (46.8%) of the preschool children were males and females, respectively. A total of 669 (14.9%) 3-year-olds, 1272 (28.3%) 4-year-olds, 1296 (28.8%) 5-year-olds and 1258 (28.0%) 6-year-olds were participated. A total of 1601 (35.6%) preschool children were Wuhan residents during Wuhan lockdown.

### Impact of oral health status

This was a multiple choice topic. During Wuhan lockdown, 519 (11.7%) pre-schoolers had oral diseases. A total of 316 (60.8%), 184 (35.5%), 95 (18.3%), 28 (5.4%), 65 (12.5%), 7 (1.4%) and 27 (5.2%) children had self-reported dental caries, toothache, halitosis, gingival bleeding, gingival swelling, tooth trauma and fillings removal, respectively.

### Impact of oral health attitudes

Because the number of participants who chose “Never” and “I don't know” was very little, we chose the data of remaining answers to analyze. Compared with that before epidemic, a greater number of caregivers would increase (rather than decrease) the attention they gave to oral health (*P* < 0.001), and they would increase their attention to their children’s oral health from 1620 (36.5%) to 2829 (63.8%) in the future (Table 1). More caregivers would increase the preventive measures they took with respect to oral health (2335, 54.2%) than decrease such measures (31, 0.7%) in the future (*P* < 0.001) (Table 2).

**Table 1:** The impact of COVID-19 on oral health attention during Wuhan lockdown and in the future (N, %)

	Oral health attention (in the future)			Total
	Increase	Decrease	No change	
Oral health attention (during)				
Increase	1411 (31.8)^a^	18 (0.4)^b^	191 (4.3)^c^	1620 (36.5)
Decrease	129 (2.9)^a^	16 (0.4)^b^	52 (1.2)^a^	197 (4.4)
No change	1289 (29.1)^a^	20 (0.5)^a^	1310 (29.5)^b^	2619 (59.0)
*P* value	0.000			
Total	2829 (63.8)	54 (1.2)	1553 (35.0)	4436 (100.0)

Unknown data were deleted

^a,b,c^Mean there were significantly different (*P* < 0.05)

**Table 2:** The impact of COVID-19 on oral health attention and preventive measures in the future (N, %)

	Preventive measures			Total
	Increase	Decrease	No change	
Oral health attention				
Increase	2118 (49.2)^a^	17 (0.4)^b^	602 (14.0)^c^	2737 (63.6)
Decrease	23 (0.5)^a^	5 (0.1)^b^	19 (0.4)^a^	47 (1.1)
No change	194 (4.5)^a^	9 (0.2)^b^	1318 (30.6)^c^	1521 (35.3)
*P* value	0.000			
Total	2335 (54.2)	31 (0.7)	1939 (45.0)	4305 (100.0)

Unknown data were deleted

^a,b,c^Mean there were significantly different (*P* < 0.05)

### Impact of oral health behaviour

#### Impact of oral hygiene behaviour

During Wuhan lockdown, there was a significant difference in oral hygiene behaviour with respect to place of residence places (*P* < 0.01). For brushing teeth, the percentage of Wuhan residents who brushed (96.9%) was higher than that of non-Wuhan residents (95.0%). For brushing frequency, 861(55.5%) Wuhan residents brushed their teeth twice a day, which was more than other residents (Table 3).

**Table 3:** Comparison of the effects of COVID-19 on the oral hygiene behaviour of preschool children (N, %)

Items	City		*P* value
	Wuhan	Others	
Brushing			0.002
Yes	1552 (96.9)^a^	2749 (95.0)^b^	
No	49 (3.1)^a^	145 (5.0)^b^	
Brushing frequency			0.000
Over twice per day	861 (55.5)^a^	1276 (46.4)^b^	
Once per day	616 (39.7)^a^	1196 (43.5)^b^	
Not everyday	75 (4.8)^a^	277 (10.1)^b^	
Brushing frequency change			0.000
Increase	147 (9.5)	292 (10.6)	
Decrease	97 (6.3)^a^	273 (10.0)^b^	
No change	1303 (84.2)^a^	2178 (79.4)^b^	
Help brushing			0.008
Everyday	432 (27.8)	708 (25.8)	
Often	384 (24.7)^a^	771 (28.0)^b^	
Occasionally	416 (26.8)	787 (28.6)	
Never	320 (20.6)^a^	483 (17.6)^b^	

^a,b^Mean there were significantly different

#### Utilization of dental services behaviour

There was a significant difference between Wuhan children and non-Wuhan children, with respect to the utilization of dental services. This included the previous dental visit, the solutions for oral diseases during Wuhan lockdown (multiple-choice topic) and, in the future, worries about the contagiousness of the disease during treatment (Tables 4 and 5).

**Table 4:** Comparison of the effects of COVID-19 on the utilization of dental services of preschool children (N, %)

Items	City		*P* value
	Wuhan	Others	
Previous dental visit (before epidemic)			0.012
Yes	176 (63.8)^a^	24 8 (54.3)^b^	
No	100 (36.2)^a^	209 (45.7)^b^	
Solutions (in the future)			0.000
Dental visit directly	584 (36.5)^a^	1507 (52.1)^b^	
Solve by themselves and have dental visit unless necessary	394 (24.6)^a^	537 (18.6)^b^	
Consultation online and have dental visit unless necessary	585 (36.5)^a^	821 (28.4)^b^	
Solve by themselves without dental visit	32 (2.0)^a^	21 (0.7)^b^	
Don’t deal with it	6 (0.4)	8 (0.3)	
Worried about contagious disease during treatment			0.004
Very worry	439 (27.8)^a^	695 (24.6)^b^	
Moderate worry	449 (28.4)	747 (26.4)	
Mild worry	518 (32.8)	1003 (35.4)	
No worry	173 (11.0)^a^	385 (13.6)^b^	
Protection during dental visit			0.000
All operations are acceptable with appropriate precautions	446 (28.5)^a^	995 (34.7)^b^	
Partial operations are acceptable with appropriate precautions	1104 (70.6)^a^	1845 (64.4)^b^	
All operations are acceptable without precautions	9 (0.6)	15 (0.5)	
Nevermind	4 (0.3)	10 (0.3)	

^a,b^Mean there were significantly different

**Table 5:** The solutions of oral diseases during Wuhan lockdown (N, %)

Items	City		*P* value
	Wuhan	Others	
Dental visit as emergency	13 (7.0)	69 (20.7)	0.000
Online consultation	32 (17.3)	38 (11.4)	0.059
Searching methods online	42 (22.7)	45 (13.5)	0.007
Taking medicine	23 (12.4)	41 (12.3)	0.958
Asking relatives and friends	13 (7.0)	29 (8.7)	0.508
Endure with observation at home	83 (44.9)	119 (35.6)	0.039

## Discussion

This survey provided insight into the impact of COVID-19 on oral health status, attitudes and behaviours. Self-reported caries prevalence during Wuhan lockdown was 60.8% and. over 30% of children suffered toothache, which might be caused by unavailable dental services [12, 13]. This showed that dentists need to pay more attention to the treatment of toothache in pre-schoolers when the epidemic were thoroughly controlled in the future.

The Fourth National Oral Health Survey reported that 50.6% and 11.6% of 3- to 5-year-old children had dental visits because of treatment and prophylaxis, respectively [4]. In this study, the results indicated that epidemics changed people’s attitudes towards oral health prophylaxis in children and they preferred to focus on the prophylaxis. Therefore, we should develop more oral health education and promotion programs providing instruction in effective prevention and the improvement of oral health [14, 15]. When the dental services are unavailable, dental professional staff can use ‘social’ digital platforms to instruct positive oral health behavioral. Through the platform, caregivers and dentist could communicate with each other and exchange information in time. Caregivers need to help preschoolers to remove food debris from cavity by brushing and using dental flossing, meanwhile, pre-schoolers can rinse to keep cavity clean after meals. They also should avoid hot/cold food to stimulate the decayed tooth or filling removed tooth. Prescription antibiotics and over-the-counter pain relievers if necessary can be taken when pre-schoolers were suffering toothache and gingival swelling. Caregivers also should take care of their children to avoid tooth trauma, if they play, protecting appliance should be used. When the restrictions were lifted, a series of non aerosol-producing procedures are recommended to minimize the risk of cross infection including chemomechanical caries removal, atraumatic restorative treatment, silver diamine fluoride, Hall Technique and so on [16, 17].

Behaviour resulted from attitude. Compared with nationwide data [18], among all pre-schoolers in this study, during Wuhan lockdown the percentage of brushing twice a day increased, moreover, the percentage of caregivers helping children brush every day increased, while the percentage of those never helping children brush obviously decreased. This result indicated that the epidemic promoted oral hygiene behaviour in preschool children. Compared to non-Wuhan children, the children in Wuhan became more active in brushing their teeth. Perhaps the differences were due to the severity of the epidemic. Wuhan was considered the center of the epidemic [19]. People there did not go out as usual, and these dental clinics were unavailable. They realized that during Wuhan lockdown only preventive measures could feasibly keep their children from developing oral diseases; they also believed that brushing teeth daily could maintain oral health [16]. Besides brushing with fluoridated toothpaste, using dental flossing and rinsing are very important to maintain oral hygiene, diet habit also should be paid attentions by caregivers. They are advised to provide fruits and vegetables, roughage and fiber to children, while avoid fermentable carbohydrates and soft drinks [20].

In light of the utilization of dental services, before the epidemic, a higher proportion of children in Wuhan attended dental visits, which indicated that they already had good utilization of dental services. However, during Wuhan lockdown, when children developed oral diseases, the majority of them had to consult online and endure their discomfort at home. The results predicted that the epidemic changed dental visit patterns. With a high risk of cross-infection, nonurgent elective appointments had to be postponed, and remote consultations and online professional solutions were recommended [21, 22]. Several studies demonstrated that patient-doctor remote clinical consultation could monitor patients’ oral health status, improve dental knowledge and reduce costs and COVID-19 dissemination [23–25]. Therefore, such consultations might be a substitution for dental visits when they were unavailable.

In this study, we found that Wuhan residents were more worried than non-Wuhan residents were. Cities in Italy, whose epidemic was once the most severe among the cities in Europe, had similar cases [26]. A third of people experienced symptoms of mild/moderate and severe distress, which were associated with worries about dying in cases of contagion [27]. Their worries were reflected in their dental visit behaviour. When individuals had oral diseases, even if in the future, they would have to make dental visits unless they could address these diseases by themselves. Moreover, most Wuhan residents elected to have only partial dental treatment performed with proper protection. Thus, in Wuhan, dental services and practices were limited due to worries about contagion in the future. It suggested we should take a series of effective measures to minimize the risk of viral cross-infection and offer a safer clinical environment for decreasing patients’ worries.

In addition to the significant contributions of the study, there are also some limitations that should be noted. Because this study was an online survey, people who did not know how to use the Internet could not participate. As with other open Internet surveys, this study also faced the challenges of respondents’ identification and information bias [28, 29]. However, the existing samples already provided a preliminary reflection of oral healthcare status in pre-schoolers and information about their challenges with dental services.

## Conclusion

Pre-schoolers’ oral health status and behaviour as well as caregivers’ attitudes were significantly influenced by the COVID-19 epidemic in China especially in Wuhan, which might give rise to a long-term impact on dental healthcare in the future. The findings suggested that we would pay more attention to pre-schoolers in Wuhan by instructing them in maintaining oral health and consulting professional advice online effectively.

## Supplementary Information

**Additional file 1.** Questionnaire in Chinese.

**Additional file 2.** Questionnaire translated into English.

## Data Availability

The datasets used and/or analyzed during the current study available from the corresponding author on reasonable request.

## Abbreviations

ECC: Early childhood caries
QR: Quick response
