# Supplementary material for: The impact of coronavirus lockdown on oral healthcare and its associated issues of pre-schoolers in China: an online cross-sectional survey
Source: BMC Oral Health. 2021 Feb 6;21:54. doi: 10.1186/s12903-021-01410-9 (PMC7865116; doi:10.1186/s12903-021-01410-9)
Supplement: Supplementary file 1 — Additional file 1. Questionnaire in Chinese. [file 12903_2021_1410_MOESM1_ESM.docx]

**武汉封城期间学龄前儿童口腔健康问卷**

亲爱的家长们：

您好！

由于新冠肺炎疫情的爆发，自2020年1月23日武汉封城开始到现在三个月以来，武汉人民的生活受到了极大的影响。为了了解疫情期间学龄前儿童口腔健康情况，为疫情后的口腔诊疗保健工作重点提供指导，武汉大学口腔医院预防科特邀您参与这次问卷调查。本次问卷采用线上自填模式，一共30题，您只需要抽出宝贵的5分钟，如实回答以下问题并提交就可以了。完成并提交这份问卷的行为即表明您已知情并同意参与此项调查。我们承诺，本次问卷您填写的所有私人信息不会被用于除了本次研究之外的任何地方，衷心感谢您的参与！

1. 孩子性别 [单选题]

○男

○女

2.孩子年龄 [单选题]

○3岁

○4岁

○5岁

○6岁

3.疫情期间，孩子所在城市 [填空题]

_________________________________

4.疫情期间孩子主要照顾者是？ [单选题]

○父母亲

○家庭长辈

○其他

5.疫情期间，您孩子的作息时间较以往是否发生改变？ [单选题]

○是

○否

○不知道

6.疫情期间，您孩子情绪波动较以往如何？ [单选题]

○增多

○减少

○没有变化

○从来没有情绪波动

○不知道

7.疫情期间，当孩子调皮时您是否会用糖果等零食来安抚他/她？ [单选题]

○是

○否

○没有调皮

8.疫情期间，您孩子进食以下食品或饮料的频率如何？[矩阵单选题]

每天≧2次 每天1次 每周2-6次 每周1次 每月1-3次 很少/从不

甜点心和糖果 ○ ○ ○ ○ ○ ○

甜饮料 ○ ○ ○ ○ ○ ○

加糖牛奶/酸奶/奶粉/茶/豆浆/咖啡 ○ ○ ○ ○ ○ ○

9.疫情期间，您孩子进食甜食或饮料的频率较以往如何？ [单选题]

○增多

○减少

○没有变化

○从不进食甜食或饮料

○不知道

10.疫情期间，您孩子刷牙吗？ [单选题]

○刷牙

○不刷 (请跳至第15题)

11.疫情期间，您孩子的刷牙频率如何？ [单选题]

○每天2次及以上

○每天1次

○不是每天刷

12.疫情期间，您孩子的刷牙频率较以往如何？ [单选题]

○增多

○减少

○没有变化

○不知道

13.疫情期间，您帮助孩子刷牙吗？ [单选题]

○每天

○有时

○偶尔

○从没做过

14.疫情期间，您孩子刷牙时用牙膏吗？ [单选题]

○是

○否

○不知道

15.疫情期间，您孩子出现全身疾病(如腹泻、感冒、手足口等)的情况较以往如何？[单选题]

○增多

○减少

○没有变化

○从来没有发生全身疾病

16.疫情期间，您对孩子口腔健康的关注程度较以往如何？ [单选题]

○增多

○减少

○没有变化

○从不关注

17. 疫情前，您孩子是否有口腔问题或不适？ [单选题]

○是

○否 (请跳至第21题)

○不知道 (请跳至第21题)

18.疫情前，您孩子有下列哪些口腔问题或不适？ [多选题]

□蛀牙

□牙痛

□口腔异味

□牙龈出血

□牙龈肿痛

□牙外伤

□充填材料脱落

□其他

19.疫情前，您小孩去医院看过牙吗？ [单选题]

○看过

○从来没看过

20.疫情期间，您孩子的上述口腔问题或不适较以往如何？ [单选题]

○加重

○减轻

○没有变化

21. 疫情期间，您孩子是否出现了口腔问题或不适？ [单选题]

○是

○否 (请跳至第25题)

○不知道 (请跳至第25题)

22.疫情期间，您孩子出现了下列哪些口腔问题或不适？ [多选题]

□蛀牙

□牙痛

□口腔异味

□牙龈出血

□牙龈肿痛

□牙外伤

□充填材料脱落

□其他

23.疫情期间，您孩子出现口腔问题或不适后您是怎么处理的？ [多选题]

□急诊或医院就医

□网络医生问诊

□网上搜索处理方法

□服用药物

□询问亲友

□忍着不做处理，居家观察

□其他

24.疫情期间，您是否因为孩子无法及时得到口腔专业诊疗而担心？ [单选题]

○非常担心

○比较担心

○有点担心

○不担心

○不知道

25.疫情结束后，您会比疫情前更加重视预防孩子的口腔疾病吗？ [单选题]

○会更重视

○没有疫情前重视

○没有变化

○从来都不重视

○不知道

26.疫情结束后，您打算给孩子采取的口腔保健措施会比疫情前 [单选题]

○增多

○减少

○没有变化

○从来都没有采取口腔保健措施

○不知道

27.疫情结束后，您是否担心因为进行口腔诊疗而感染传染病？ [单选题]

○非常担心

○比较担心

○有点担心

○不担心

○不知道

28.疫情结束后，如果您孩子遇到口腔问题，您会如何做？ [单选题]

○去医院就诊，积极寻求医生的帮助

○先自行想办法解决，解决不了再去医院

○先行线上问诊，必要时再去医院

○不去医院，自行想办法解决 (请跳至第30题)

○不去医院，也不做任何处理 (请跳至第30题)

29.疫情结束后，如果您带孩子到医院看牙，您认为孩子 [单选题]

○只要适当防护，就可以接受所有口腔预防和治疗的操作 (请跳至第问卷末尾，提交答卷)

○只要适当防护，就可以接受部分口腔预防和治疗的操作 (请跳至第问卷末尾，提交答卷)

○无需防护，就可以接受所有口腔预防和治疗的操作 (请跳至第问卷末尾，提交答卷)

○无所谓 (请跳至第问卷末尾，提交答卷)

30.疫情结束后，如果您孩子遇到口腔问题，您也不带孩子去医院看牙的原因是？ [多选题]

□乳牙要替换，不需要换

□经济困难，看不起牙

□太忙、没有时间

□害怕因看牙感染传染病

□孩子害怕看牙疼痛

□其他
