# Supplementary material for: The impact of coronavirus lockdown on oral healthcare and its associated issues of pre-schoolers in China: an online cross-sectional survey
Source: BMC Oral Health. 2021 Feb 6;21:54. doi: 10.1186/s12903-021-01410-9 (PMC7865116; doi:10.1186/s12903-021-01410-9)
Supplement: Supplementary file 2 — Additional file 2. Questionnaire translated into English. [file 12903_2021_1410_MOESM2_ESM.docx]

**Oral health questionnaire for pre-schoolers during Wuhan lockdown**

Dear caregivers,

As a result of the COVID-19 outbreak, the lives of people in Wuhan have been greatly affected since Wuhan lockdown on January 23, 2020. In order to understand the oral health status of preschool children during the epidemic period and provide guidance for oral health care after the epidemic, preventive dentistry department of Stomatology Hospital of Wuhan University invites you to participate in this questionnaire survey. This questionnaire adopts online self-filling mode, with a total of 30 questions. It will take you about 5 minutes to answer the following questions truthfully. The questionnaire is completed on a voluntary basis. Completing and submitting this questionnaire would be regarded as consent to participate. We promise that all the personal information you fill in this questionnaire will not be used anywhere except this study. Thank you for your participation!

1. Child gender [single choice question]

1. Male
2. Female

2. Age of children [single choice question]

A. 3 years old

B. 4 years old

C. 5 years old

D. 6 years old

3. During Wuhan lockdown, which city did the child live in? [fill in the blank]

_________________________________

4. Who was the main caregivers of children during Wuhan lockdown? [single choice question]

A. Parent

B. Grandparent

C. Other

5. Did your child's time schedule change during Wuhan lockdown? [single choice question]

A. Yes

B. No

C. I don't know

6. Compared with before, how was your child's mood fluctuation during Wuhan lockdown? [single choice question]

A. Increase

B. Decrease

C. No change

D. Never had mood fluctuation

E. I don't know

7. During Wuhan lockdown, when the child was naughty, did you comfort him / her with snacks such as candy? [single choice question]

A. Yes

B. No

C. No mishief

8.1 How often did your child eat desserts and candy during Wuhan lockdown? [matrix choice question]

A. More than twice per day

B. Once pre day

C. 2-6 times pre week

D. Once pre week

E. 1-3 times pre month

F. Rarely / Never

8.2 How often did your child drink sweet beverages during Wuhan lockdown? [matrix choice question]

A. More than twice per day

B. Once pre day

C. 2-6 times pre week

D. Once pre week

E. 1-3 times pre month

F. Rarely / Never

8.3 How often did your child eat sweetened milk / yogurt / milk powder / tea / soybean milk / coffee during Wuhan lockdown? [matrix choice question]

A. More than twice per day

B. Once pre day

C. 2-6 times pre week

D. Once pre week

E. 1-3 times pre month

F. Rarely / Never

9. How often did your child consume sweets or drinks during the epidemic? [single choice question]

1. Increase
2. Decrease
3. No change
4. Never consume sweets or drinks
5. I don't know

10. Did your child brush teeth during Wuhan lockdown? [single choice question]

1. Yes
2. No (please skip to question 15)

11. How often did your child brush teeth during Wuhan lockdown? [single choice question]

A. Over twice per day

B. Once per day

C. Not everyday

12. How did the frequency of your child brush teeth during Wuhan lockdown? [single choice question]

1. Increase

B. Decrease

C. No change

D. I don't know

13. Did you help your child brush teeth during Wuhan lockdown? [single choice question]

A. Everyday

1. Often

C. Occasionally

D. Never

14. During Wuhan lockdown, did your child use toothpaste when brushing his teeth? [single choice question]

A. Yes

B. No

C. I don't know

15. During Wuhan lockdown, how did your child suffer from systemic diseases (such as diarrhea, cold, hand, foot and mouth, etc.)? [single choice question]

A. Increase

B. Decrease

C. No change

D. Never had a systemic illness

16. Compared with before, how did your attention change to child's oral health during Wuhan lockdown? [single choice question]

1. Increase

B. Decrease

C. No change

D. Never pay attention

17. Before Wuhan lockdown, did your child have oral problems or discomfort? [single choice question]

A. Yes

B. No (please skip to question 21)

C. I don't know (please skip to question 21)

18. Before Wuhan lockdown, which of the following oral problems or discomfort did your child have? [multiple choice questions]

A. Tooth decay

B. Toothache

C. Bad breath

D. Gingival bleeding

E. Gingival swelling and pain

F. Tooth trauma

G. The filling material loss

H. Others

19. Before Wuhan lockdown, did your child have dental visit? [single choice question]

A. Yes

B. No

20. During Wuhan lockdown, how did your child's oral problems or discomfort compared with the past? [single choice question]

A. Aggravation

B. Mitigation

C. No change

21. Did your child have oral problems or discomfort during Wuhan lockdown? [single choice question]

A. Yes

B. No (please skip to question 25)

C. I don't know (please skip to question 25)

22. Which of the following oral problems or discomfort did your child have during Wuhan lockdown? [multiple choice question]

A. Tooth decay

1. Toothache
2. Bad breath
3. Gingival bleeding
4. Gingival swelling and pain
5. Tooth trauma
6. The filling material loss
7. Others

23. How did you deal with your child's oral problems or discomfort during Wuhan lockdown? [multiple choice questions]

A. Dental visit as emergency

B. Online consultation

C. Searching method online

D. Taking medicine

E. Asking relatives and friends

F. Enduring with observation at home

G. Others

24. During Wuhan lockdown, how did you worry that your child could not get professional dental treatment in time? [single choice question]

A. Very worry

B. Moderate worry

C. Mild worry

D. No worry

E. I don't know

25. Compared with before, how did your attention change to child's oral health after Wuhan lockdown? [single choice question]

A. Increase

B. Decrease

C. No change

D. Never pay attention

E. I don't know

26. Compared with before, how did the oral health care preventive measures you plan to take for your child after Wuhan lockdown? [single choice question]

A. Increase

B. Decrease

C. No change

D. Never take oral care measures

E. I don't know

27. After Wuhan lockdown, how will you worry about contagious disease during dental treatment? [single choice question]

A. Very worry

B. Moderate worry

C. Mild worry

D. No worry

E. I don't know

28. What will you do if your child have oral problems after Wuhan lockdown? [single choice question]

A. Dental visit directly

B. Solve by themselves and have dental visit unless necessary

C. Consultation online and have dental visit unless necessary

D. Solve by themselves without dental visit (please skip to question 30)

E. Don’t deal with it (please skip to question 30)

29. After Wuhan lockdown, if you take your child to dental visit, how do you think about the protection? [single choice question] (please skip to the end and submit the questionnaire)

A. All operations are acceptable with appropriate precautions

B. Partial operations are acceptable with appropriate precautions

C. All operations are acceptable without precautions

D. Nevermind

30. After Wuhan lockdown, if your child has oral problems, why do you not take your child to the hospital for dental care? [multiple choice questions]

A. The deciduous teeth will be replaced and don’t need to be treated

B. Economic difficulties

C. Too busy, no time

D. Be afraid of cross-infection due to dental visit

E. Children’s dental anxiety

F. Others
